# Adaptation of the Nursing Activities Score in Latvia

**DOI:** 10.3390/ijerph21101284

**Published:** 2024-09-26

**Authors:** Olga Cerela-Boltunova, Inga Millere, Ingrida Trups-Kalne

**Affiliations:** 1 Department of Nursing and Midwifery, Faculty of Health and Sports Sciences, Riga Stradiņš University, LV-1067 Riga, Latvia; 2 Psychology Laboratory, Institute of Public Health, Riga Stradiņš University, LV-1067 Riga, Latvia

**Keywords:** Nursing Activities Score, nurse workload, content validity, intensive care, Latvia, adaptation

## Abstract

This study focuses on the adaptation and validation of the Nursing Activities Score (NAS) for use in Latvian intensive care units (ICUs) to measure nursing workload. The NAS, widely used internationally, was selected for its comprehensive ability to reflect 81% of ICU nursing activities, making it a suitable tool for assessing nursing workload in the Latvian healthcare context. The study followed a two-phase methodology: (1) expert validation using the Content Validity Index (CVI) and (2) a pilot study to assess the psychometric properties of the adapted tool. In the first phase, 10 ICU nursing experts assessed the translated NAS items, resulting in revisions to three specific paragraphs (4a, 14, and 20) based on low CVI scores. After refinement, CVIs improved from 0.6 to 0.8 for paragraphs 4a and 14, and from 0.5 to 0.9 for paragraph 20. The final CVI for all items reached 0.909. In the second phase, a pilot study was conducted in a Latvian ICU with 42 patients and 226 NAS assessments. The psychometric evaluation showed strong reliability and validity, confirming the NAS’s suitability for measuring nursing workload in this context. Cronbach’s alpha for the scale was 0.973. The adapted NAS provides a standardised method for workload assessment in Latvian ICUs, offering potential improvements in nurse resource management and patient care.

## 1. Introduction

The work environment of nursing personnel has changed considerably due to several factors, including the reform of the healthcare system, restructuring of hospitals, and personnel shortages [1]. These changes have promoted higher stress among nurses who refer to their work environment as the main source of stress [1,2]. Several studies [3,4,5,6] conducted at intensive care wards emphasise the importance of evaluating the workload of nurses, which is difficult due to the qualitative character of nursing work. Despite the importance of workload, the literature lacks a unified, clear definition of workload. However, it has been recognised as an essential factor that affects our well-being and career decisions. The resolution of workload-related issues is essential for maintaining a sustainable and supportive work environment for nursing care specialists [7].

Excessive workload in nursing has several detrimental effects on both nurses and patient outcomes. High levels of workload can lead to burnout, emotional exhaustion, and job dissatisfaction among nurses, which significantly increases turnover rates [8]. When nurses are consistently overburdened, they are more likely to experience stress and fatigue, contributing to a decline in their overall well-being and job performance [2,9]. This often results in higher absenteeism, reduced job retention, and increased turnover, further exacerbating staffing shortages in healthcare facilities [10].

From a patient care perspective, an excessive nursing workload is associated with adverse outcomes such as medication errors, delayed treatments, and lapses in monitoring [11]. Nurses under pressure may struggle to maintain high standards of care, leading to a higher likelihood of complications, infections, or even mortality [12]. Insufficient staffing and overworked nurses can also impact the timeliness and quality of communication with patients and their families, which is crucial in preventing misunderstandings and ensuring effective care [13]. Ultimately, the strain on nurses not only affects their health and job satisfaction but also compromises patient safety and the overall quality of healthcare services [12,13].

The workload of nurses may be calculated as the time and physical and cognitive effort that nurses are required to exert in order to implement direct and indirect actions and non-patient-related care operations [14]. It is a complex, multilateral concept that the nurses need to assess, with consideration of the factors that determine additional patient care needs in relation to organisation, the work of the department, teamwork, the individual and the system of care in general [15].

While analysing the number of personnel resources and planning the increase of personnel, healthcare managers need substantiation for the re-organisation of nursing positions at the structural unit [16]. This entails explanations of whether the number of nursing personnel was higher than required by the effective laws and regulations, or, on the contrary, the provision of personnel failed to comply with the workload required to provide care [15]. The lack of qualified medical personnel has sparked worldwide discussions on the workload of nurses. The topicality of this issue is high to date because a significant incompatibility between the development of medical technologies and the workload faced by intensive care unit nurses still exists [17]. To measure this, a specific tool is required, which can determine the situation at hand [15].

Measurements of nursing workload were first commenced in the 1970s. The initial TISS—Therapeutic Intervention Scoring System [18]—has been upgraded several times and the version that is currently in use worldwide is TISS-28 [19]. Later, in 2003, the TISS-28 research team adapted components to reflect more than the severity of the disease alone and developed the Nursing Activities Score—NAS, which reflects the diverse character of the nursing workload [15].

Measuring workload in intensive therapy wards is essential due to the high complexity of care, the need for constant vigilance, and the direct link between staffing levels and patient outcomes [20]. Unlike general nursing specialties, ICU nursing involves more intense, time-sensitive care that requires specialised skills, lower patient-nurse ratios, and proactive management of nurse fatigue and burnout [21]. Proper workload measurement in ICUs is critical to ensuring patient safety, nurse well-being, and the efficient allocation of resources [20,21].

The NAS is considered a better tool for measuring nursing workload than others due to several reasons. One of the primary advantages is its comprehensive coverage of nursing activities [22,23,24,25]. Unlike other tools such as the TISS-28, Time-Oriented Score System (TOSS), or Nine Equivalents of Nursing Manpower Use Score (NEMS), which focuses mainly on therapeutic interventions, NAS includes both direct patient care and indirect activities such as administrative tasks and family support [26,27]. This broader scope provides a more accurate reflection of the workload in intensive care units. Another reason is that NAS offers increased accuracy in measuring time spent on nursing activities. It accounts for a larger percentage of the nursing time dedicated to patient care compared to TISS-28, as it considers not only medical interventions but also routine care and necessary administrative work [21]. NAS has also been validated through studies in multiple countries and is used worldwide, making it a reliable tool for various healthcare settings [3,4,5,6,21]. Additionally, NAS has proven to be practical and feasible to apply, with a clear structure that aligns well with everyday nursing tasks [26]. This makes it an effective tool for health managers to assess and allocate resources efficiently, which is crucial in high-demand environments like intensive care units. These factors contribute to NAS being a widely adopted and preferred tool for nursing workload measurement over others.

The NAS was chosen as the most suitable tool for the Latvian healthcare context due to its widespread use and proven reliability in measuring nursing workload, particularly in ICUs. The NAS provides a detailed and comprehensive assessment of nursing activities, including direct patient care and indirect activities [15], which aligns well with the demands of Latvian healthcare, where systematic workload measurement has not yet been implemented.

Nursing managers, hospital managers and politicians, in their articles, interviews and conference speeches, often refer to the shortage of nurses and the workload of nurses, and they talk about the fact that nurses need to care for larger numbers of patients than they are physically capable of doing; however, few studies have measured the above phenomena, as well as there being no preventive measures to address this problem of the healthcare system [28,29]. Latvian ICUs, like many in Eastern Europe, face challenges such as high nurse-patient ratios and increasing demands on ICU nurses [30]. By using NAS, healthcare managers in Latvia could gain a clearer understanding of the actual workload faced by nurses, allowing for better distribution of tasks and more informed decisions about staffing levels [31]. This tool can also support the alignment of resources with patient care needs, ensuring that nurses are neither overburdened nor underutilized, which directly contributes to improving patient outcomes. Additionally, by incorporating NAS data into management practices, Latvian healthcare facilities could optimise nurse-to-patient ratios [15], address inefficiencies, and enhance the quality of care in a way that is responsive to the unique demands of the Latvian healthcare system [32].

Studies from countries with similar healthcare environments, such as Belgium [21], have shown that high nurse workload is associated with increased turnover rates and negative patient outcomes. Although no specific studies have been conducted in Latvia, reports from ICUs highlight similar challenges, emphasising the need for systematic workload assessment.

Most studies dealing with the workload of nurses worldwide have been conducted at intensive therapy wards [5,6,11,20,21,22,23,24,33]. This study addresses that gap by providing data specific to the Latvian healthcare system, which has not been previously examined. By focusing on this under-researched region, the study not only adds to the broader understanding of nursing workload but also offers a valuable comparison with international findings. This localised insight can help inform policy decisions and improve resource management in Latvia’s healthcare institutions [34,35].

The research suggests that the Nursing Activities Score covers 81% of nursing activities at the intensive care ward, while TISS-28 only covers 43.3%. Therefore, NAS was specifically selected for adaptation and further research [21,27,36]. The results of the score do not depend on the severity of the disease, the combination of cases or the type of intensive care ward. This tool enables the standardised use of the score at units for clinical, as well as research purposes [15] and is appropriate to measure nursing activities.

The Score was developed by Miranda D.R., Nap R., Rijik De A., Schaufeli W., Iaoichino G. and the TISS tool work group in 2003 [15,37].

On 29 October 2022, a reply from the professor at the University of Groningen (The Netherlands), D.R. Miranda, was received electronically. In the received e-mail message, the professor gave permission to use the Nursing Activities Score that was developed in 2003 in Latvia. A description of how to use the scale and interpret results was also provided.

## 2. Materials and Methods

### 2.1. Description of the Tool

The Nursing Activities Score tool includes the following subcategories: monitoring and titration; hygiene procedures; mobilisation and positioning; support and care for the patient, as well as their family; administrative and management tasks [20].

The initial 2003 version of the above tool [15] offers 7 principal categories and 23 statements or actions. In accordance with the developed questionnaire, 18 statements have been provided with a binary choice, while 5 statements (points 1, 4 and 6–8) have answer options with points granted for one option only (a, b or c). The weight of the points reflects the percentage of time that one nurse spends on patient care for the performance of the indicated action if such has been required. Five care actions with answer options are marked with an asterisk [15].

In accordance with the instruction, a nurse is required to fill out the tool once for 24 h or twice for 12 h actions per patient. Every 24 or 12 h, a nurse shall fill out a separate nursing assessment tool for each patient. It is important to do it at the same time every day, for instance, at 7:00 a.m. If the patient has been discharged from an intensive care unit but the bed place was taken by another patient within the same 12 h or 24 h interval, the results of the Nursing Activities Score for both patients shall not be summed up, and the assessment shall be performed for each patient individually [15].

If the tool is filled out twice within a period of 24 h (for the day shift and for the night shift), the tool for the activities performed during the day shall be filled out at 7:00 p.m., which reflects the need of the patients for nursing care activities during the day, while nursing activities performed during the night shift must be registered at 7:00 a.m., before the commencement of the next shift. When filling out the Nursing Activities Score, a nurse must mark whether they performed the particular actions, where the answer is yes, or they did not perform the activities, where the answer is no. By counting the total of actions performed by the nurse, the number of points is obtained [15]. 

Each activity has a score; therefore, the total score is obtained by summing the points granted to the patient, which complies with direct and indirect patient care needs. This parameter demonstrates how much nursing time was paid to the patient over the last 12 or 24 h. Thus, if the score is 100 points, it is believed that the patient has received 100% of the time of the nurse for their care over the last 12 or 24 h. This parameter may reach a maximum of 176.8% per patient [19,21,38]. In general, one point corresponds to 14.4 min [39].

The sum of results for 23 statements ranges from 0% to 176.8% (or the approximate equivalent of 1.8 full-time nursing workloads over a period of 12 or 24 h). A result that exceeds 100 points (100%) demonstrates that the required patient care can only be provided by several nurses at the same time. If the obtained result is 50%, the nurse-to-patient ratio must be 1:2; results starting from 100 mean 100% of care time, which translates into a ratio of 1:1, while results that exceed 100% mean that more than one nurse is required to provide care to the particular patient, at a ratio of 2:1 [15].

### 2.2. Method

This is a methodological study aimed at the inspection of the content of the tool and the validity thereof by expert agreement and in a focus group.

During the translation process, it was initially determined that the NAS would be translated from English into the official language of Latvia—Latvian. The translation process began with the selection of a unidirectional translation strategy, when one independent translator from an official translation agency translated the original scale. After the translation was completed, the survey was sent to the original author of the NAS for final review and approval to ensure that the translated version accurately reflected the content and intent of the original scale. The original author, proficient in both English and Latvian, reviewed and confirmed that the translation was accurate.

First phase—determination of the content validity index (CVI)—a type of validity check that determines the degree of expert evaluation consensus regarding the statements. Ten experts participated in the first phase.

Criteria for the inclusion of experts—nurse, head nurse or deputy head nurse with a certificate in “anaesthesia and intensive care nursing” with at least 5 years experience in an intensive care ward, education—a Master’s degree in healthcare.

Second phase—considering the fact that statements with a CVI below 0.78 were present [40], a focus group consisting of 10 experts was assembled. The focus group’s objective, in accordance with the Delphi method, was to check the validity of the content [41]. The focus group was held in the form of a video conference of a remote online focus group meeting. Phase 1 and Phase 2 had the same 10 experts.

During the second phase of adaptation, the focus group was organised in a safe ZOOM environment guaranteeing anonymity, which was inaccessible to unauthorised personnel [42]. During the focus group session, an assistant was employed who made notes of the interaction that occurred in the focus group. A moderator of the focus group was one of the article’s authors who holds certification in anesthesia and intensive care nursing and has 9 years experience working in the ICU. The total duration of the focus group session, including a break, was 1 h and 36 min.

During the focus group session, three paragraphs (No. 4.a., No. 14, and No. 20) that needed to be reviewed were indicated to the experts. During the discussion, each of the three paragraphs was reviewed individually. The experts were encouraged to express their opinions on the wording, clarity, and relevance of each paragraph in the context of Latvian ICU practices. These discussions included comparing different wording suggestions, considering cultural and clinical contexts, and ensuring medical terminology was accurate. The experts debated various formulations and reached a common agreement on the final wording for each paragraph.

After the focus group, the CVIs for the three revised paragraphs were re-evaluated, again involving the same 10 experts.

The aim of the pilot study was to evaluate the psychometric properties, including reliability and validity, of the adapted NAS within the Latvian ICU context. The pilot study aimed to determine whether the adapted NAS accurately reflects nursing workload and to identify any necessary refinements in its use for measuring direct and indirect patient care activities in ICUs.

The validity criteria for the pilot studies phase required participants to be registered nurses. Survey participants had to complete all sections of the scale, be over 18 years old, work in ICU, have at least 3 months work experience in the field, and hold certification in “anesthesia and intensive care nursing”.

The pilot study was performed at one of the intensive therapy wards of Latvia in the time period from 10 November 2022 to 11 December 2022. Data collection involved nurses filling out the NAS for each patient in the ICU, once every 12 h, using a digital tool.

**Figure 1:**
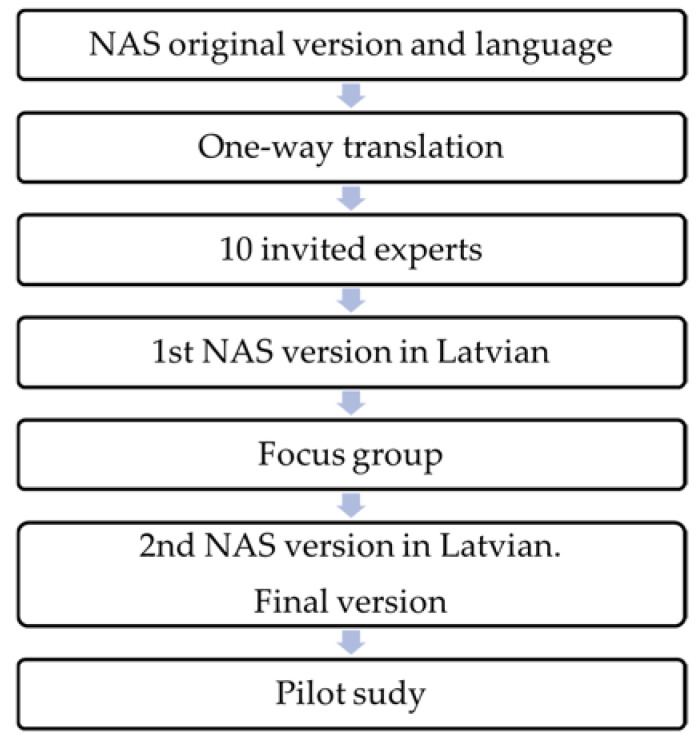
Adaptation process of Nursing Activities Score.

The process of adaptation is reflected in Figure 1.

### 2.3. Ethical Considerations

The ethical considerations for this study included obtaining informed consent from all participating nurses and experts, ensuring that they were fully informed about the study’s purpose, procedures, and their right to withdraw at any time. Confidentiality was maintained by anonymising all personal data, following data protection regulations. The study received ethical approval from the Ethics Committee of Riga Stradiņš University (protocol code 2-PĒK-4/416/2023), ensuring adherence to ethical standards.

Efforts were made to minimise the burden on participants, particularly ICU nurses, to prevent any additional stress or interference with patient care. Transparency was maintained by disclosing that there were no conflicts of interest. Finally, the study aimed to improve nursing workload management in Latvia, ensuring that the research aligned with the principles of beneficence and non-maleficence by promoting positive outcomes for both nurses and patients without causing harm.

Due to the observational nature of the study and the anonymisation of the data, the written consent of patients or relatives was not required.

## 3. Results

Ten experts participated in the first and second phases of adaptation, with the average age of experts 49 years. All experts were women, with 8 of them having work experience of more than 10 years. The experts’ experience included direct care in work with patients, research, knowledge transfer in work with students, as well as management experience.

A translated tool was sent to each of the experts (1st version of the tool). The objective of this phase was to review statements in order to check whether the researched questions were completely represented and complied with the objectives and tasks formulated within the study and to check the technical execution of the questionnaire by finding out whether it contained unclear, “suggestive” or repetitive statements in terms of content. Each expert had to evaluate the compliance of each paragraph with the concept of the Nursing Activities Score, where 0 means does not comply, and 1 means complies. The results are represented in Table 1.

According to Lynn [41], it has been determined that if the number of experts is equal to or higher than 9, the CVI may not be lower than 0.78. If the CVI is lower than 0.78, the provided paragraphs or statements are incorrect and need to be reviewed or rephrased.

After the first phase of the study, it was established that the total CVI for all paragraphs was 0.874; the total CVI for all experts was 0.871. The CVI for all experts varied from 0.78 to 0.97. It was generally detected that in paragraphs No. 4.a (CVI–0.6), No. 14 (CVI–0.6), and in No. 20. (CVI–0.5), the CVI was lower than 0.78.

After the discussion during the second phase, the moderator of the focus group summarised all the data. Ideas and paragraph formulations were considered, as a result of which the final version of the tool in Latvian was accepted by the focus group.

A total of three paragraphs were edited. Paragraph No. 4.a: “Performance of hygiene procedures, for instance, redressing of wounds and intravascular catheters, underwear change, washing of the patient in the event of incontinence, vomiting, care for burn wounds, purulent wounds, care for complex post-surgical wounds with drains, performance of special procedures (for instance, care for the patient in a sterile environment to prevent infection, cleaning of rooms after infections, personnel hygiene), etc.” was edited to “Performance of hygiene procedures, for instance, redressing of wounds and intravascular catheters, underwear change, washing of the patient, care for burn wounds, purulent wounds, care for complex post-surgical wounds with drains, performance of special procedures (cleaning of rooms after infections, personnel hygiene), etc.”. Paragraph No.4a. was reworded for clarity and simplicity, removing redundant details.

Paragraph No. 14: “Left ventricle monitoring. Pulmonary artery catheter with or without measurements of heart ejection function parameters” was edited to “Monitoring of cardiovascular system, PAP monitoring”. Paragraph No.14. was adjusted with clearer terminology to better reflect Latvian ICU practices.

Paragraph No. 20: “Intravenous hyperalimentation” was edited to “Total parenteral nutrition”. Paragraph No.20. was revised with more accessible language, aligning with common clinical terminology in Latvia.

After the focus group, the CVIs for the three revised paragraphs were re-evaluated. For paragraph No. 4.a., the CVI was initially 0.6, and after revision, it improved to 0.8; for paragraph No. 14., the CVI was 0.6, and after revision, it increased to 0.8; for paragraph No. 20, the CVI was 0.5, and after revision, it became 0.9. After the paragraphs were re-evaluated, it was established that the total CVI for all paragraphs was 0.897; the total CVI for all experts was 0.897. The CVI for all experts varied from 0.82 to 0.97.

After the discussion and review of the paragraphs by the focus group, the 2nd tool version in Latvia was obtained—the last version. The final edition of all the paragraphs is reflected in Table 2. Adaptation of the content is marked in italics. In both phases, changes to the results and individual items were introduced, which would change the structure of the Nursing Activities Score.

### 3.1. Pilot Study—Phase 1

When the final version of the Nursing Activities Score scale in Latvian was developed, the first pilot study was conducted, during which 5 intensive care nurses who participated in the pilot study with the purpose of obtaining face validity were surveyed. Face validity refers to the extent a concept is reflected by the content of a tool that is intended to measure the respective concept [43].

While measuring face validity, the five nurses were asked the following question: “Did you understand what each paragraph was about when you filled out the Nursing Activities Score?”. Each nurse had to evaluate the compliance of each paragraph with the concept of the Nursing Activities Score, where 0 means does not comply, and 1 means complies. The results are represented in Table 3.

While measuring face validity, the conclusion was drawn that the FVI is 0.90, which shows evidence of high validity and safety [44].

### 3.2. Pilot Study—Phase 2

In the pilot study, a total of 17 nurses from one Adult ICU participated. The average age of the nurses was 46 years (range 23–64), and all participants were female. The nurses had an average of 13 years of professional experience (range 2–21 years), with 64.7% holding a bachelor’s degree in nursing. In this phase of the study, nurses completed scales based on their experience with real ICU patients. However, for this research, patient-specific information was not required. The only patient-related data collected was the number of patients in the Adult ICU during the one-month period in which the pilot study was conducted.

During the aforementioned period in the Section 2, the Nursing Activities Score was filled out 226 times regarding 42 patients.

During the pilot study, the nurses filled out a total of 109 scores during the night shift and 117 scores during the day shift.

The intensive care unit in question has six bed places. The percentage of bed capacity is reflected in Table 4.

Considering the capacity of beds, it can be determined that beds 1, 2, and 3 are used most often. This fact can be explained by the proximity of the aforementioned beds to the nurses’ post.

The objective of the pilot study was to obtain the psychometric parameters of each paragraph (mean, SD, min. and max, corrected item-total correlation, Cronbach’s alpha) while performing the descriptive statistics. The psychometric parameters of each paragraph can be seen in Table 5.

Most of the items demonstrate high corrected item-total correlations, with values exceeding 0.75, indicating that they significantly enhance the internal consistency [44] of the scale. Items such as 1.a (0.961), 7.a (0.988), 8.c (0.987), and 17 (0.982) exhibit exceptionally strong correlations, showing a robust alignment with the total score. Since all correlations are positive, each item contributes positively to the scale without detracting from its overall effectiveness.

The overall Cronbach’s alpha is 0.973, indicating excellent internal consistency. Additionally, removing any individual item does not substantially improve this value, as the Cronbach’s alpha if item deleted remains near 0.973. This suggests that each item is integral to the scale’s reliability. Items 17 and 18, in particular, are crucial, as their removal would slightly reduce the alpha to 0.972.

During the day, two nurses provide patient care in the ICU involved in the pilot study. The results suggest that 70.14% of one nurse’s time over 24 h is occupied with patient care. Data analysis shows eight night shifts and nine day shifts during which the required number of nurses exceeded two based on patient activities.

Within the framework of the pilot study, it was found that most often—98.7% of the time—nurses report being involved in the receipt of medications by the patient, followed by short-term consultations provided to the relatives of the patient—97.8%, and the measurement of patient diuresis—95.1%. In general, these activities are the principal standard nursing care activities while providing patient care. The nurses noted that they performed the following patient care activities less frequently: patient hygiene for longer than 4 h—0.9%, and relative support for longer than 3 h—2.2%.

## 4. Discussion

This study aimed to adapt and validate the NAS for use in Latvian ICUs. The significance of the NAS lies in its ability to accurately measure nursing workload [3], which is crucial for enhancing nursing practice and improving patient care outcomes in Latvia.

In Phase 1, the expert validation process involved a group of experienced ICU nurses who evaluated the relevance and clarity of the NAS items. The resulting high CVI confirms [41] that the tool accurately reflects the specific nursing activities relevant to the Latvian context. This expert input not only enhances the cultural appropriateness of the NAS but also ensures that it aligns with the practical realities of nursing in Latvia.

The implications of these findings are substantial. By confirming the validity of the NAS, the study paves the way for its adoption as a reliable tool for assessing nursing workload. This can lead to better workload management [45], as nursing managers will have access to data that accurately represent the demands placed on nursing staff. Consequently, this may facilitate more effective staffing decisions, ultimately contributing to improved job satisfaction and retention among nurses, as well as enhanced patient care [2,7,9,20].

In Phase 2, the pilot study demonstrated robust reliability, with a Cronbach’s alpha of 0.973, indicating excellent internal consistency of the adapted NAS. These findings are critical as they affirm that the NAS can be reliably used to measure nursing activities in the ICU setting. The pilot study also highlighted how the adapted NAS can capture both direct and indirect nursing activities, thereby providing a more comprehensive understanding of nursing workload.

The implications of these findings extend to both clinical practice and policy-making in healthcare. For nursing practice, the ability to accurately measure workload through the NAS means that healthcare providers can identify specific areas where nurses may be overburdened [46]. This insight allows for targeted interventions, such as redistributing tasks or adjusting staffing levels based on real-time workload data [47]. Consequently, the adoption of the NAS can lead to a reduction in nurse burnout [48] and improve the overall quality of patient care.

From a policy perspective, the findings support the need for systemic changes in how nursing workloads are assessed and managed in Latvia. The validated NAS can serve as a benchmark for future research and practice, providing a foundation for national guidelines that promote optimal staffing levels and resource allocation in ICUs [49]. As healthcare demands continue to grow, implementing tools like the NAS will be essential for ensuring that nursing resources are utilised effectively and that patient safety is prioritised [11,14,50].

In our study, a total workload value of 70.14% (SD = 5.31) was established in the pilot study, close to the 2016 study [51] and a study conducted in Brazil [52] (66.5% (SD = 9.1)). European researchers described average tool values as 65.9% (SD = 6.6) and 65.97% (SD = 2.53) in Spain [53,54]. According to global research [51,52,53,54], overall scores ranged from 50.4% to 96.2%.

Our study did not assess concurrent validity between NAS and another tool due to the lack of a validated instrument for measuring ICU nurses’ workload in this specific setting. However, similar research has validated NAS by comparing it with other tools, such as TISS-28 or other standardised workload assessment instruments [4,6].

The clinical implications of implementing NAS in Latvia require further exploration, particularly regarding how it can improve nursing practice compared to other workload tools. Unlike TISS-28 or NEMS, which focus on clinical interventions and patient severity [26,27], NAS offers a more holistic evaluation by including direct patient care and indirect nursing tasks [21]. This comprehensive approach helps ensure a more equitable workload distribution and reduces stress and burnout [2,9,14,20].

The adoption of the Latvian version of the NAS has substantial clinical significance for several reasons. First and foremost, accurate measurement of nursing workload is essential for ensuring adequate staffing levels, which directly impacts patient safety and quality of care [25]. According to studies, higher nurse-to-patient ratios are associated with better patient outcomes, including reduced mortality rates and fewer medication errors [49,55,56]. By utilising the NAS, healthcare managers in Latvia can make data-driven decisions regarding staffing, thereby optimising nurse allocation based on actual workload demands rather than assumptions or generalisations.

Furthermore, the NAS provides a comprehensive framework for assessing both direct patient care and indirect activities [15], such as administrative tasks and family support. This broader perspective on nursing workload acknowledges the multifaceted nature of nursing responsibilities, aligning with findings from other research that emphasises the importance of recognising all aspects of nursing work [7]. By capturing the full scope of nursing activities, the NAS not only helps in identifying areas of overwork but also highlights tasks that may be undervalued in current workload assessments, leading to more equitable work distribution [21].

Additionally, the validated NAS can serve as a critical tool for monitoring nurse well-being and preventing burnout [57]. The literature indicates that excessive workload and lack of support can lead to high levels of stress [9,53] and turnover [8,58] among nurses. The ability to measure workload effectively allows nursing leaders to implement targeted interventions, such as staff training or support systems, aimed at alleviating stressors identified through NAS assessments [59]. By addressing these issues, healthcare facilities can foster a more supportive environment that enhances job satisfaction [58,60] and retention [61] among nursing staff.

The introduction of the NAS in Latvia also aligns with global trends [33,62] advocating standardised measures of nursing workload. The World Health Organisation emphasizes [63,64] the need for evidence-based approaches to health workforce planning, suggesting that tools like the NAS can contribute to a more sustainable healthcare system. By adopting a validated workload measurement tool, Latvia positions itself to engage in international discussions on nursing practices and to contribute to the growing body of evidence supporting the importance of nursing care in improving health outcomes.

In summary, in current situations, excessive workload in Latvian ICUs contributes to nurse burnout [65] and high turnover rates [66]. NAS provides a more objective and transparent system for staffing decisions, leading to improved care quality and better patient outcomes [11,33]. High nurse-to-patient ratios in ICUs negatively affect care quality [31]. NAS standardizes care across hospitals, ensuring equitable care through objective data rather than subjective opinions [67]. The clinical significance of adopting the Latvian version of the NAS lies in its potential to improve staffing decisions, enhance patient safety, and promote nurse well-being.

## 5. Conclusions

The study successfully adapted the NAS for use in Latvia, providing a valuable tool for measuring nursing workload in intensive care units. Based on the results, the NAS proved effective in capturing a comprehensive range of nursing activities, which can significantly aid in workload management and resource allocation. The findings demonstrate that the NAS offers a more accurate and nuanced measurement of nursing workload compared to previously used tools, making it highly suitable for the Latvian healthcare context.

By providing clear insights into the distribution of nursing tasks, the NAS can help healthcare managers address issues related to nurse burnout, optimise staffing levels, and improve overall patient care. This is particularly important given the increasing demands on nurses in intensive care settings. The implementation of NAS will enable data-driven decisions that can enhance the efficiency and quality of care provided in Latvian ICUs. Overall, the adaptation of NAS in Latvia is a critical step toward improving nursing practices and patient outcomes, justifying its utility and relevance in the local healthcare system.

In addressing the limitations, this study acknowledges the constraints of a single-centre design and a limited sample size, which may affect the generalisability of the results. Additionally, the expert selection process could introduce bias, highlighting the need for further research in diverse clinical settings to fully evaluate the NAS’s applicability across different contexts.

### Limitations

The limitations of the study include several factors that could influence the generalisability and interpretation of the results. The pilot study was conducted in a single ICU in Latvia with a limited number of patients (42) and assessments (226), which may not fully represent the diversity of ICU settings and nursing practices across the country. This single-centre design restricts the external validity of the findings, as differences in patient populations, staff workloads, and ICU protocols in other hospitals may affect the applicability of the adapted NAS. Additionally, although ten experienced ICU nursing experts participated in the tool’s content validation, the relatively small and specific group may have provided a narrow perspective, limiting the generalisability of the findings to a wider range of clinical environments.

The study also focused solely on ICU settings, meaning the NAS adaptation’s applicability to other hospital departments, such as general wards or step-down units, was not tested. Further research would be necessary to explore its use in these contexts. While the psychometric properties of the adapted NAS were evaluated, the study relied on short-term data collection. Longitudinal studies with larger datasets may be required to fully assess the tool’s reliability and validity over time. The study did not account for the impact of different levels of technology, staffing, or resource availability across various ICUs, which could also influence how the NAS is applied and its effectiveness in measuring workload.

Furthermore, the methods employed in the study—use of the content validity index (CVI) and focus group discussions—have inherent limitations. The CVI provides a superficial assessment of how well items measure the intended construct and may not fully capture deeper, more complex conceptual relationships. Similarly, face validity, while useful, offers only a limited evaluation of the tool’s effectiveness. Focus group discussions, though helpful for refining the tool, are susceptible to participant bias, and the views expressed may not fully represent the broader perspectives of the nursing population. These methodological limitations should be taken into account when interpreting the study results, as they may influence the perceived accuracy and applicability of the adapted NAS in real-world healthcare settings.

By acknowledging these limitations, future research can address these issues to further refine and validate the NAS for broader application in Latvia and other healthcare settings.

## Figures and Tables

**Table 1:** Expert evaluation of item validity.

Items	Expert 1.	Expert 2.	Expert 3.	Expert 4.	Expert 5.	Expert 6.	Expert 7.	Expert 8.	Expert 9.	Expert 10.	CVI	
1.a.	1	1	1	1	1	1	1	1	1	1	1.0	
1.b.	1	1	1	0	1	1	1	1	0	1	0.8	
1.c.	1	1	1	1	1	0	1	1	1	1	0.9	
2.	1	1	1	1	1	1	1	1	1	1	1.0	
3.	1	1	1	1	1	1	1	1	1	1	1.0	
4.a.	0	1	1	1	0	1	0	1	0	1	0.6	
4.b.	0	1	1	1	1	1	1	1	1	1	0.9	
4.c.	0	1	1	1	1	1	1	1	1	1	0.9	
5.	1	1	1	1	1	1	1	1	1	1	1.0	
6.a.	1	1	0	1	1	1	1	1	0	1	0.8	
6.b.	1	1	1	1	0	0	1	1	1	1	0.8	
6.c.	1	1	1	1	1	1	1	1	1	1	1	
7.a.	1	1	1	0	1	1	1	1	0	1	0.8	
7.b.	1	1	1	1	0	1	1	1	1	0	0.8	
8.a.	1	1	1	1	1	0	1	1	1	1	0.9	
8.b.	1	1	0	1	1	1	1	0	1	1	0.8	
8.c.	1	1	0	1	1	1	1	0	1	1	0.8	
9.	1	1	1	1	0	1	1	1	1	1	0.9	
10.	1	1	1	1	1	1	1	1	1	1	1	
11.	1	1	1	1	1	1	1	1	1	1	1	
12.	1	1	1	1	1	1	1	1	1	1	1	
13.	1	0	1	1	1	1	1	1	0	1	0.8	
14.	1	1	0	0	1	1	0	1	0	1	0.6	
15.	1	1	0	1	1	1	0	1	1	1	0.8	
16.	1	1	1	1	1	1	1	1	1	1	1.0	
17.	1	1	1	1	1	1	1	1	1	1	1.0	
18.	1	0	1	1	1	1	1	1	1	1	0.9	
19.	1	1	0	1	1	1	1	1	1	1	0.9	
20.	1	0	0	0	0	1	1	1	0	1	0.5	
21.	1	0	1	1	1	1	1	1	1	1	0.9	
22.	1	1	1	1	0	0	1	1	1	1	0.8	
23.	1	1	1	1	1	1	1	1	1	1	1.0	
CVI	0.91	0.88	0.78	0.88	0.81	0.88	0.91	0.94	0.78	0.97		0.87
											0.87	

**Table 2:** Final version of the Nursing Activities Score in Latvian.

Items	Activities	Yes	No	Score
1.a	Monitoring and titration * Recording of vital signs once per hour and calculation of fluid balance.			
1.b	* The nurse is with the patient and observes them or actively interacts with the patient ≥2 h per shift due to the severe health condition of the patient or the specific nature of the therapy. For instance, if the patient requires non-invasive mechanical ventilation, if the ventilatory support needs to be gradually withdrawn, if the patient is anxious, or disorientated, if the patient needs to lie in a prone position in order to take test samples, or if the patient needs to be prepared for the injection of fluid and/or other medications.			
1.c	* The nurse is with the patient and observes them or actively interacts with the patient ≥ 4 h per shift due to the severe health condition of the patient or the specific nature of the therapy, considering the aforementioned examples.			
2.	Additional tests, biochemical and microbiological examinations.			
3.	Medications: excluding vasoactive medications.			
4.a	* Performance of hygiene procedures, for instance, redressing of wounds and intravascular catheters, underwear change, washing of the patient, care for burn wounds, purulent wounds, care for complex post-surgical wounds with drains, performance of special procedures (cleaning of rooms after infections, personnel hygiene) etc.			
4.b	* Performance of patient hygiene, long >2 h per shift.			
4.c	* Performance of patient hygiene, long >4 h per shift.			
5.	Care for all drains, except gastric probe.			
6.a	Mobilisation and positioning of the patient, including the turning of the patient; patient mobilisation by moving the patient from the bed into the chair/wheelchair; lifting of the patient with the help of other employees (for instance, immobile patient, dragging, prone position). * Performance of a procedure once every eight hours.			
6.b	* Performance of a procedure more frequently than once every eight hours or by 2 nurses.			
6.c	* Performance of a procedure by ≥3 nurses (and frequently).			
7.a	Provision of support to relatives and the patient, including calls, questioning and consulting. * Provision of support to relatives and patient care lasts for approximately one hour per a shift, for instance, their clinical status is explained, the nurse talks to the patient to reduce anxiety, discusses difficulties and the condition of the family.			
7.b	* Provision of support to relatives and patient care lasts for a total of 3 or more hours per shift, for instance, their clinical status is explained, the nurse talks to the patient to reduce anxiety, discusses difficulties and the condition of the family.			
8.a	Administrative and management duties * Performance of daily duties, for instance, processing of clinical data, referrals to examinations, transfer of information to the next shift (for instance, when personnel that arrive for their shift at the department changes).			
8.b	* Performance of administrative and management duties, which approximately lasts for a total of 2 h per shift, for instance, research, protocols used, admissions and discharges.			
8.c	* Performance of administrative and management duties, which approximately lasts for a total of ≥4 h per shift, for instance, registration of a case of death, organ donation procedures, harmonisation of activities with physicians/departments of other specialisation.			
9.	Assisted ventilation: any type of mechanical/assisted ventilation with or without positive expiratory pressure, with or without muscle relaxants; spontaneous respiration with positive expiratory pressure (for instance, CPAP or biphasic positive airway pressure (BiPAP)), with or without endotracheal intubation; supply of additional oxygen in any way.			
10.	Care for artificial airways–endotracheal tube or tracheostomy.			
11.	Therapy for the improvement of lung function: physiotherapy of the thorax, stimulating spirometry, inhalation therapy, intratracheal suction.			
12.	Any type and dosage of vasoactive medications.			
13.	Intravenous infusion of the required fluid, if large amounts of fluid have been lost. Fluid infusion > 3 L/m^2^/day irrespective of the type of infused fluid.			
14.	*Monitoring of cardiovascular system, PAP monitoring.*			
15.	Indirect cardiac massage and artificial respiration after cardiac arrest; during the last eight hours (excluding a single blow of the fist in the area of the heart).			
16.	Haemofiltration method. Dialysis method.			
17.	Measurements of the excreted amount of urine (for instance, with the use of a permanent urine catheter).			
18.	Measurements of intracranial pressure.			
19.	Therapy of complex metabolic acidosis/alkalosis.			
20.	*Total parenteral nutrition.*			
21.	Enteral nutrition: through a gastric probe or another gastrointestinal probe (for instance, via a jejunostomy).			
22.	Specific activities at the intensive care unit. Endotracheal intubation, insertion of an artificial cardiac pacemaker, cardioversion, endoscopies, emergency surgeries during the last eight hours, gastric lavage without the inclusion of regular manipulations and examinations without a direct impact on the clinical condition of the patient, for instance, X-Ray examination, echography, electrocardiogram, bandaging, or the insertion of venous or arterial catheters.			
23.	Specific activities outside the intensive care unit. Surgical operations or diagnostic measures.			

**Table 3:** Face validity (FVI)—assessment of paragraphs by nurses.

Items	Nurse 1.	Nurse 2.	Nurse 3.	Nurse 4.	Nurse 5.	FVI
1.a.	1	1	1	1	1	1.0
1.b.	1	1	1	0	1	0.8
1.c.	1	1	1	1	1	1.0
2.	1	1	1	1	1	1.0
3.	1	1	1	1	1	1.0
4.a.	0	1	1	1	1	0.8
4.b.	0	1	1	1	1	0.8
4.c.	0	1	1	1	1	0.8
5.	1	1	1	1	1	1.0
6.a.	1	1	0	1	1	0.8
6.b.	1	1	1	1	1	1.0
6.c.	1	1	1	1	1	1.0
7.a.	1	1	1	0	1	0.8
7.b.	1	1	1	1	0	0.8
8.a.	1	1	1	1	1	1.0
8.b.	1	1	0	1	1	0.8
8.c.	1	1	0	1	1	0.8
9.	1	1	1	1	0	0.8
10.	1	1	1	1	1	1.0
11.	1	1	1	1	1	1.0
12.	1	1	1	1	1	1.0
13.	1	0	1	1	1	0.8
14.	1	1	0	1	1	0.8
15.	1	1	0	1	1	0.8
16.	1	1	1	1	1	1.0
17.	1	1	1	1	1	1.0
18.	1	0	1	1	1	0.8
19.	1	1	0	1	1	0.8
20.	1	1	1	1	1	1.0
21.	1	1	1	1	1	1.0
22.	1	1	1	1	0	0.8
23.	1	1	1	1	1	1.0
FVI	0.91	0.94	0.81	0.94	0.91	0.9
						0.9

**Table 4:** Bed capacity.

Number of the Bed	Frequency	%
1	51	22.6
2	39	17.3
3	43	19.0
4	31	13.7
5	32	14.2
6	30	13.3
Total number	226	100.0

**Table 5:** Psychometric parameters of the paragraph.

Items	M	SD	Min	Max	Corrected Item-Total Correlation	Cronbach’s Alpha If Item Deleted
1.a.	2.05	0.15	0.0	4.5	0.961	0.974
1.b.	6.16	0.40	0.0	12.1	0.958	0.973
1.c.	0.69	0.24	0.0	19.6	0.859	0.973
2.	1.39	0.13	0.0	4.3	0.750	0.977
3.	5.53	0.04	0.0	5.6	0.758	0.976
4.a.	2.30	0.14	0.0	4.1	0.913	0.973
4.b.	7.08	0.55	0.0	16.5	0.878	0.973
4.c.	0.18	0.13	0.0	20	0.898	0.973
5.	1.42	0.05	0.0	1.8	0.729	0.976
6.a.	2.36	0.18	0.0	5.5	0.874	0.975
6.b.	6.69	0.41	0.0	12.4	0.764	0.974
6.c.	1.35	0.31	0.0	17	0.859	0.974
7.a.	3.91	0.04	0.0	4	0.988	0.973
7.b.	0.71	0.31	0.0	32	0.856	0.973
8.a.	3.40	0.11	0.0	4.2	0.875	0.974
8.b.	4.21	0.60	0.0	23.2	0.901	0.974
8.c.	0.80	0.32	0.0	30	0.987	0.974
9.	0.71	0.05	0.0	1.4	0.738	0.975
10.	1.26	0.06	0.0	1.8	0.859	0.974
11.	1.89	0.15	0.0	4.4	0.814	0.974
12.	0.94	0.03	0.0	1.2	0.900	0.973
13.	0.85	0.08	0.0	2.5	0.875	0.973
14.	0.26	0.04	0.0	1.7	0.929	0.973
15.	0.69	0.14	0.0	7.1	0.957	0.973
16.	2.62	0.24	0.0	7.7	0.915	0.973
17.	6.66	0.10	0.0	7	0.982	0.972
18.	0.08	0.02	0.0	1.6	0.975	0.972
19.	0.27	0.04	0.0	1.3	0.952	0.973
20.	0.79	0.08	0.0	2.8	0.939	0.973
21.	1.04	0.04	0.0	1.3	0.957	0.973
22.	1.14	0.09	0.0	2.8	0.940	0.973
23.	0.71	0.06	0.0	1.9	0.957	0.973
Total Value of Workload	70.14	5.31	0.0	176.8		0.973

## Data Availability

The datasets produced and examined in this study can be obtained from the corresponding author upon reasonable request. All data generated or analysed during this study are provided within the published article. The data utilised in this study are confidential.
